# Exploring the Genetic Resistance to Gastrointestinal Nematodes Infection in Goat Using RNA-Sequencing

**DOI:** 10.3390/ijms18040751

**Published:** 2017-04-01

**Authors:** Ali Akbar Bhuiyan, Jingjin Li, Zhenyang Wu, Pan Ni, Adeyinka Abiola Adetula, Haiyan Wang, Cheng Zhang, Xiaohui Tang, Anjuman Ara Bhuyan, Shuhong Zhao, Xiaoyong Du

**Affiliations:** 1 Key Laboratory of Agricultural Animal Genetics, Breeding and Reproduction, Ministry of Education, College of Animal Science and Veterinary Medicine, Huazhong Agricultural University, Wuhan 430070, China; aab76_blri@yahoo.com (A.A.B.); jingjinli@webmail.hzau.edu (J.L.); wuzhenyang0724@gmail.com (Z.W.); nipanmato@gmail.com (P.N.); quueenyk10@yahoo.com (A.A.A.); shzhao@mail.hzau.edu.cn (S.Z.); 2 College of Informatics, Huazhong Agricultural University, Wuhan 430070, China; info_blri@yahoo.com (H.W.); aabso_blri@yahoo.com (C.Z.); 3 College of Agricultural Animal Husbandry, Tibet University, Linzhi 850012, China; s_azmal@yahoo.com; 4 State Key Laboratory of Agricultural Microbiology, Ministry of Education, College of Animal Science and Veterinary Medicine, Huazhong Agricultural University, Wuhan 430070, China; aab_bau@yahoo.com; 5 Senior Scientific Officer, Bangladesh Livestock Research Institute, Savar, Dhaka-1341, Bangladesh

**Keywords:** transcriptome, genetic resistant, gastrointestinal nematodes, RNA-seq, goat

## Abstract

Gastrointestinal nematodes (GINs) are one of the most economically important parasites of small ruminants and a major animal health concern in many regions of the world. However, the molecular mechanisms of the host response to GIN infections in goat are still little known. In this study, two genetically distinct goat populations, one relatively resistant and the other susceptible to GIN infections, were identified in *Yichang* goat and then four individuals in each group were chosen to compare mRNA expression profiles using RNA-seq. Field experiment showed lower worm burden, delayed and reduced egg production in the relatively resistant group than the susceptible group. The analysis of RNA-seq showed that 2369 genes, 1407 of which were up-regulated and 962 down-regulated, were significantly (*p* < 0.001) differentially expressed between these two groups. Functional annotation of the 298 genes more highly expressed in the resistant group yielded a total of 46 significant (*p* < 0.05) functional annotation clusters including 31 genes (9 in innate immunity, 13 in immunity, and 9 in innate immune response) related to immune biosynthetic process as well as transforming growth factor (TGF)-β, mitogen-activated protein kinase (MAPK), and cell adhesion molecules (CAMs) pathways. Our findings provide insights that are immediately relevant for the improvement of host resistance to GIN infections and which will make it possible to know the mechanisms underlying the resistance of goats to GIN infections.

## 1. Introduction

Gastrointestinal nematode (GIN) infections are well known as a major animal health concern, mainly affecting grazing livestock worldwide, especially in developing countries. The main problem to efficient chevon production is GIN infections which therefore, represents global per capita protein unavailability. A serious impact on weight gain in small ruminants is due to GIN infections, which can contribute to decreases in weight gain of about 14% [1]. Live weight loss and severe fall in PCV (Packed Cell Volume) can be caused by *Haemonchus contortus*, the main prevalent nematode in humid and semi-humid regions, also known as haematophagous parasites [2]. Breeding studies of small ruminants have revealed a reduction in egg per gram (EPG) feces, with a concurrent selective breeding of naturally resistant small ruminants to GIN infection [3,4]. This continuous corroboration to enhanced resistance is frequently performed on kids to breed goats that are more resistant to GIN infection [5,6]. There is increasing evidence for genetic variation in resistance to GINs in sheep and goats [7,8]. Additive genetic variation is the main consideration in variation among all the factors related to determining EPG variation, and it accounts for about 30% of the variation in EPG [9]. Heritability of matured worm length in small ruminant is high, ranging from 0.62 to 0.68 [9], while medium levels of heritability are observed for the EPG trait, varying from low-medium (0.14) to high medium (0.33) [10]. Using demographic data, fecal egg count (FEC) and body weight records of sheep population, Hayward et al. [11] found positive selection of susceptibility of GIN infections, where on average animals that slowly decreased body weight with rising burden of parasites had higher breeding success across the whole generation. Some genetic mapping studies have identified quantitative trait loci (QTL) regions in caprine genomes demonstrating animal-specific variation in resistance to nematode infection in sheep [12,13,14]. Pathogen-induced phenotypic changes were always concurrent with remarkable alterations in gene expression in hosts. The existing animal-specific variations in the acquired immune response against *Haemonchus* contortus have been associated with increasing a competent Th-2 biased immunity response driven through the interleukin-4, interleukin-5, and interleukin-13 cytokines in sheep [15,16]. Innate immune components are also likely involved in activities of anti-nematode responses in resistant goats. For example, lectins were up-regulated by interleukin-4 after worm infection [17]. A weakness side of the candidate gene approach is that a multifactorial and multigenic basis of resistance/susceptibility to GIN suggests that the regulatory context of transcription factors plays an important role in anti-nematode responses. In order to gain a better understanding of cellular functions resulting from interactions between goat and GINs, it would be helpful to have knowledge of gene expression at the global level. In this study, we identified two genetically distinct *Yichang* white goat populations (one relatively resistant to GINs, the other susceptible), and compared mRNA profiles using RNA-seq. Our results indicate that the involvement of inducible immune components, including protein kinase activity, complement activation, as well as transforming growth factor (TGF)-β, mitogen-activated protein kinase (MAPK), and cell adhesion molecules (CAMs) pathways, play significant roles in the resistance of goats to GIN infection.

## 2. Results

### 2.1. Egg Per Gram (EPG), Weight Gain, and Blood Parameters during Browsing Period

**Figure 1:**
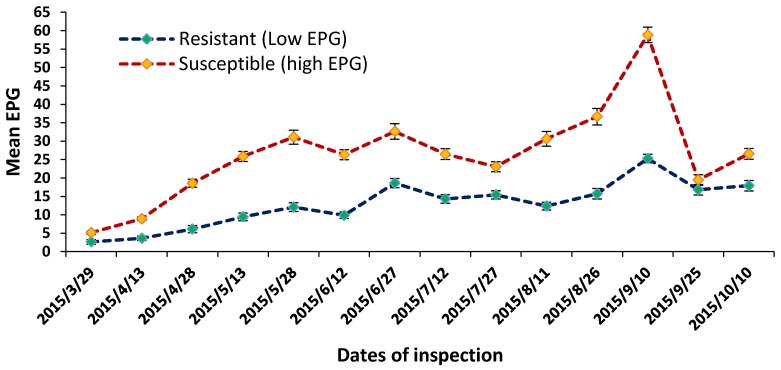
Fortnightly mean egg per gram (EPG) in feces are shown in resistant (low EPG, *n* = 48) and susceptible (high EPG, *n* = 74) groups of *Yichang* White Goats (YWGs). The *X*-axis represents dates of inspection, the *Y*-axis represents mean EPG, and error bars represent standard error of mean (SEM).

The average fortnightly (two weeks) EPG values (Mean ± SEM) were 11.43 ± 1.81 and 42.26 ± 6.56 for resistant (low EPG) and susceptible (high EPG) groups of *Yichang* White Goats (YWGs), respectively (*n* = 122), during the seven month browsing period (Table 1). In all of the browsing period except 29 March 2015 and 25 September 2015, the resistant group consistently had lower EPG values (*p* < 0.01) than the EPG susceptible group (Figure 1). Furthermore, over the browsing period, the resistant group gained more live-weight (*p* < 0.01) than the susceptible group (34.75 ± 2.65 vs. 20.16 ± 2.38 g/day).

We measured hematological parameters every alternate month during the browsing period and found that susceptible goats had significantly lower hemoglobin (*p* < 0.01) and lower PCV percentage (*p* < 0.05) compared to resistant goats (Table 1). It was also shown that susceptible goats had lower hemoglobin than their normal hemoglobin value (90–150 g/L). There were no significant differences between red blood cells (RBCs), white blood cells (WBC), lymphocytes, monocytes, and granulocytes.

Based on the above phenotypic data, we selected four high resistant individuals from the resistant group and four susceptible individuals from the susceptible group, respectably. After the nematode challenge trial, the mean number of total *Haemonchus contortus* worms were recovered from resistant goats (1129 ± 44) was still significantly less (*p* < 0.05) than those of susceptible goats (2194 ± 84).

### 2.2. General Characteristics of the Goat Peripheral Blood Transcriptome

We used next generation sequencing to analyze the expression profiles of resistant and susceptible YWGs following GIN infections. A total of 8 RNA-seq libraries were unambiguously constructed by mRNA sequencing. The sequencing quality related parameters are shown in Table 2 and Figure S1. An average of 35.22 ± 5.5 (mean ± SD) million reads was generated per sample. The distribution of GC percentage (i.e., the BP ratio of G and C through the reads), the presence or absence of over-represented sequences, and the skewed position of the histogram from the supplementary figure (Figure S1) proved that the sequence quality was good. The ratio of the properly matched reads of alignment ranged from 78.68% to 84.9% in resistant goats and from 87.64% to 89.91% in susceptible goats. The reads alignment to goat genome indicated that the sequencing reads were of good quality and the sequencing depth was sufficient for a differential expression analysis between the two groups of these goats. The analysis of RNA-seq data showed that 2369 genes were significantly (*p* < 0.001) differentially expressed between these two groups, where 1407 were up-regulated and 962 were down-regulated genes (see National Center for Biotechnology Information (NCBI) Sequence Read Archive (SRA); Bio Project ID: PRJNA 341785).

### 2.3. Differentially Expressed Genes (DEGs) of Resistant and Susceptible Yichang White Goats (YWGs)

**Figure 2:**
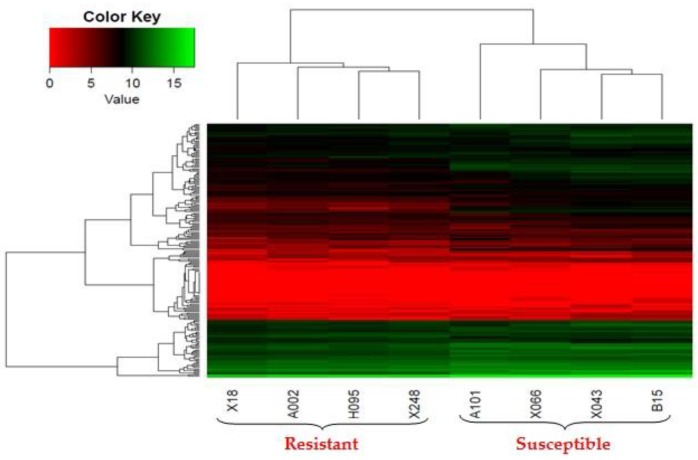
Differentially expressed genes (DEGs) between resistant and susceptible YWGs. Heatmap of standardized reads-count in eight samples of resistant and susceptible goats. Each row represents genes and the column represents samples (resistant and susceptible goat IDs), and the color key values (0 to 15 scale) shows sequentially minimum to maximum expression.

A heatmap illustrating values of the mostly differentially expressed 200 genes between the resistant and the susceptible YWGs is shown in Figure 2. Each row represents DEGs, the column represents samples (goat IDs), and color key represents the expression levels of DEGs, where the 0 to 15 scale shows sequentially minimum to maximum expression rate in the heatmap. Finally, 298 mostly DEGs were taken on the basis of their fold change (FC) values in relation to their expression levels (up-regulated FC (log_2_) ≥ 2.5 and down-regulated FC (log_2_) ≤ −2.0), with significantly different expression levels between the resistant and susceptible goats. Among these selected DEGs, 151 were highly expressed in susceptible goats and 147 were expressed in resistant goats.

### 2.4. Gene Ontology (GO)

Out of the 298 DEGs (FC ≥ 2.5 and ≤ −2.0), 282 orthologues of human genes were annotated in the DAVID (Database for Annotation, Visualization and Integrated Discovery) database. Functional annotation classification of expressed genes in the susceptible and resistant goats produced 46 significant (*p* < 0.05) functional annotation clusters, including 31 genes (9 in innate immunity, 13 in immunity, and 9 in innate immune response) related to the immune biosynthetic process. The most significant clusters are listed in Table 3. The functional annotation clusters in the resistant group related to the immunity, innate immunity, and innate immune response *biosynthetic process* whereas negative regulation of transcription from RNA polymerase II promoter, protein phosphorylation, and GTPase activation processes were related in the susceptible group.

**Figure 3:**
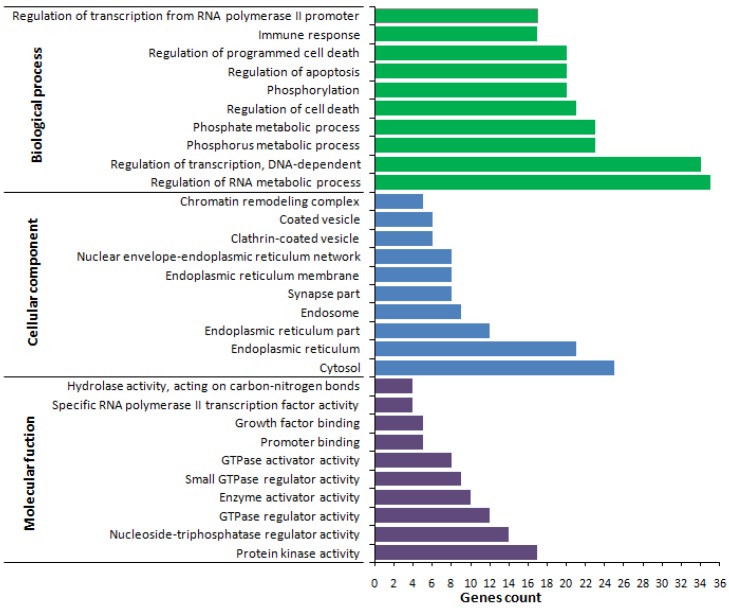
The ten most enriched gene ontology (functional classifications) of differentially expressed genes (DEGs) between resistant and susceptible YWGs.

The functional classification of DEGs in the biological processes (BPs), cellular components (CCs), and molecular functions (MFs) were annotated on the basis of Gene Ontology (GO) categories. Under this classification, 106 BP categories (201 genes), 19 CC categories (175 genes), and 13 MF categories (206 genes) were annotated using DAVID classification analysis. The 10 most enriched categories of DEGs in the GO functional classification are shown in Figure 3. Under the BP category, large numbers of genes were categorized as regulation of RNA metabolic process (35 genes), and regulation of the transcription-DNA dependent (34 genes) and phosphorus metabolic process (23 genes). Under the CC category, cytosol (25 genes), endoplasmic reticulum (21 genes), and endoplasmic reticulum part (12 genes) represented the highest proportion of genes. Under the MF category, protein kinase activity (17 genes), nucleoside-triphosphatase regulator activity (14 genes), and GTPase regulator activity (12 genes) were the most abundant sub-categories (Figure 3 and Table S1).

For the up-regulated genes, enriched biological process GO terms related to the immune system, included complement C3 precursor, complement component 3, C-type lectin domain family 4, integrin α4, ST6 β-galactosamide α-2, and 6-sialyltranferase-1; whereas, leukocyte specific transcript 1, interleukin 1 receptor, type II, interferon-induced protein 44-like, and α-inducible protein 6 were over-represented among down-regulated genes (Figure 3 and Table S1).

### 2.5. Signaling Pathway Analysis of the DEGs

Based on conserved orthologues defined by Kyoto Encyclopedia of Genes and Genomes (KEGG), 151 of the up-regulated and 147 of the down-regulated genes branded in the susceptible animals were assigned to one or more conserved biological sub-pathways. The sub-pathways in KEGG and related genes (count = 2, EASE = 0.5) were listed in Table 4 and Table S2. This analysis identified 23 sub-pathways, including those in endocytosis, Huntington’s disease, MAPK signaling pathway, chronic myeloid leukemia, adherens junction, hypertrophic cardiomyopathy (HCM), and TGF-β signaling pathway, that showed abundant genes in sub-pathways (Table 4 and Table S2).

Pathways of chronic myeloid leukemia, adherens junction, apoptosis, and endocytosis were enriched in up-regulated genes, except IQ motif and Sec7 domain 2 (*IQSEC2*) genes in pathways in endocytosis. Hypertrophic cardio-myopathy, Huntington’s disease, Parkinson’s disease, and MAPK signaling pathways were enriched for both up- and down-regulated genes, while TGF-β signaling pathway and cell adhesion molecules were enriched in mostly up-regulated genes.

### 2.6. Genetic Association Disease Classes of DEGs of Resistant and Susceptible YWGs

**Figure 4:**
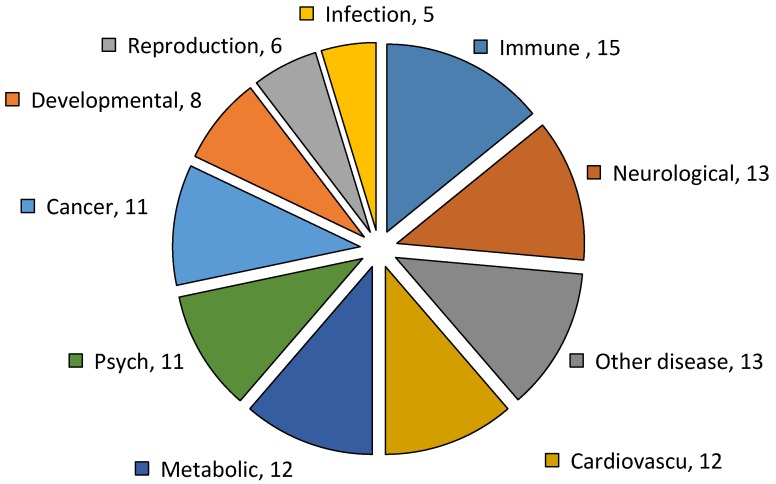
The pie chart of the 10 most abundant genetic association disease classes of differentially expressed genes (DEGs) showing in the parentheses the number of genes that differed between resistant and susceptible YWGs.

Fifteen genetic associated disease classes were identified from the 298 DEGs in the DAVID analysis (count = 2, EASE = 1). The pie chart of gene ontology (GO) on 10 most abundant genetic association disease classes of DEGs between resistant and susceptible YWGs in relation to GIN infections are shown in Figure 4 and Figure S2. The most abundant genetic associated disease classes were immune response genes (15 genes), followed by chronologically neurological (13 genes), cardiovascular (12 genes), metabolic (12 genes), psych (11 genes), and cancer (11 genes). The immune response genes were particularly associated with genetic resistance and susceptibility to a wide array of diseases. Among the immune response genes of DEGs between resistant and susceptible YWGs on GIN infections, seven genes (*ATP7A*, *BCL2*, *ITGA4*, *ST6GAL1*, *CMKLR1*, *ERAP1*, and *C3*) were up-regulated and eight genes (*CLEC4E*, *CCL27*, *F12*, *IFI6*, *IFI44L*, *IL1R2*, *LST1*, and *PGLYRP1*) were down-regulated in resistant and susceptible goats (Table 5 and Figure S2).

We specifically investigated our differential transcriptional outcomes for genes identified as being involved in immune responses (Figure 4 and Figure S2). Notable genes up-regulated in susceptible goats included *BCL2*, *CD96*, *ITGA4*, and *ST6GAL1*. Some of the down-regulated DEGs involved in immune response system (i.e., *CLEC4E*, *CCL27*, *F12*, *IFI6*, *IFI44L*, *LST1*, and *PGLYRP1*) had been sorted in susceptible goats. These genes were usually recognized as indicators of t-cell responses [18]. Few interleukins-related genes were found to be transcribed in this study, namely *IL10BP*, *IL6R*, *IL1R1*, and *IL1R2*.

### 2.7. DEGs Confirmation by qRT-PCR

**Figure 5:**
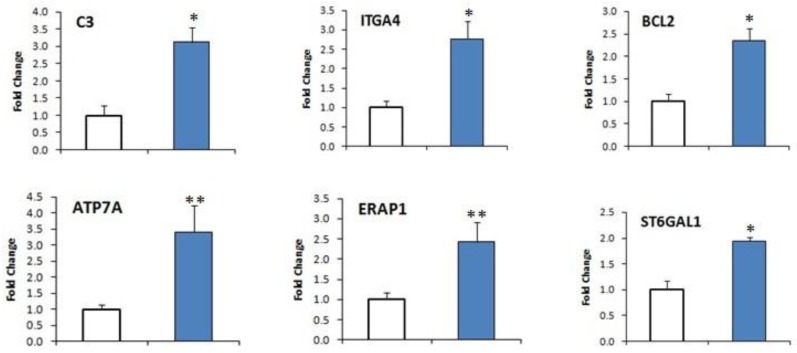

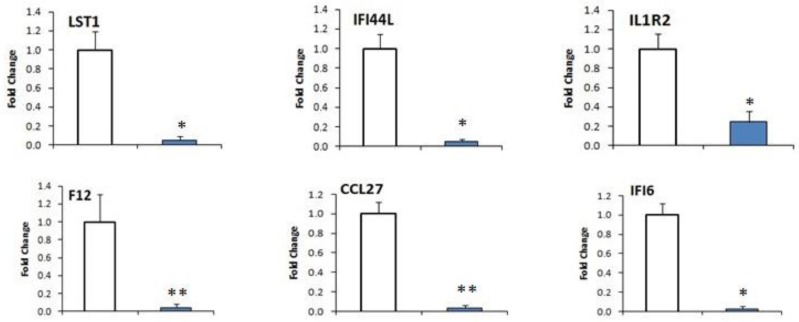
Validation of DEGs by Quantitative Reverse-Transcriptase Polymerase Chain Reaction (qRT-PCR) with RNA sequence results between resistant and susceptible goats. Clear bars represent the resistant goats and the blue bars represent susceptible goats. All samples were normalized by β-actin housekeeping gene. Among the selected 12 genes, 6 genes (*C3*, *ITGA4*, *BCL2*, *ATP7A*, *ERAP1*, and *ST6GAP1*) were highly expressed in susceptible goats and another 6 genes (*LST1*, *IFI44L*, *IL1R2*, *F12*, *CCL27*, and *IFI6*) were lower expressed in susceptible goats compared with resistant goats. * Significant at *p* < 0.01 and ** significant at *p* < 0.05, based on assuming unequal variances Student’s *t*-test. Error bars represent standard error of the mean (SEM).

A total of 12 DEGs (six up-regulated and six down-regulated genes) were selected from Table 5 for Quantitative Reverse Transcriptase Polymerase Chain Reaction (qRT-PCR) examination to validate the expression profile obtained from RNA sequencing. All samples were normalized by β-actin, a housekeeping gene. Among the selected genes, six up-regulated genes (*C3*, *ITGA4*, *BCL2*, *ATP7A*, *ERAP1*, and *ST6GAP1*) were higher expressed in susceptible goats and another six down-regulated genes (*LST1*, *IFI44L*, *IL1R2*, *F12*, *CCL27*, and *IFI6*) were lesser expressed in susceptible goats, compared with resistant goats (Figure 5 and Figure S3). Differentially expressed genes *C3*, *ITGA4*, *BCL2*, *ST6GAL1*, *LST1*, *IFI44L*, *IL1R2*, and *IFI6* were significantly different (at *p* < 0.01 levels) and *ATP7A*, *ERAP1*, *F12*, and *CCL27* were significantly different (at *p* < 0.05), as expected from the RNA sequenced data.

**Figure 6:**
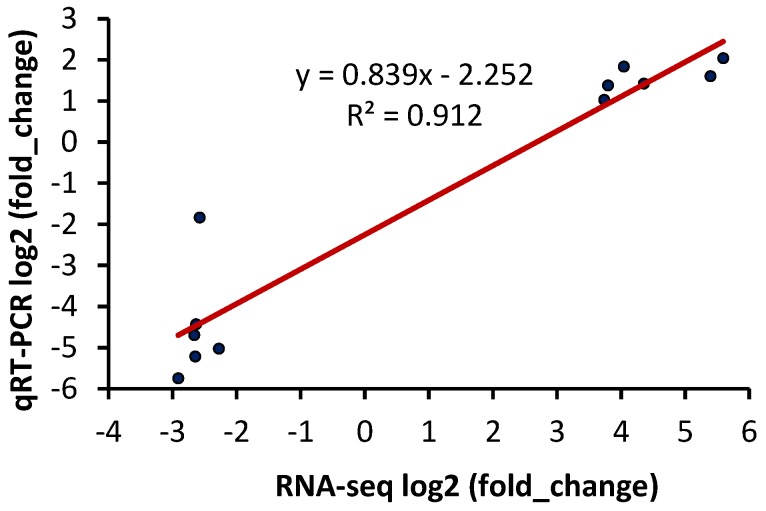
Line fit plot of qRT-PCR log2 (fold change) data and mRNA sequence log2 (fold change) data of selected differentially expressed genes between resistant and susceptible goats showing trend line, R-squared value, and linear regression model.

The qRT-PCR log2 (fold-change) data and mRNA sequence log2 (fold-change) data were fitted in the line plot and expression pattern. As expected from the line fit plot, the selected gene expression patterns of resistant and susceptible goats were validated by qRT-PCR and were strongly correlated with the sequencing results (correlation coefficient is 0.912, and linear regression model is *Y* = 0.839*X* − 2.252) (Figure 6 and Figure S4).

## 3. Discussion

GIN infections are one of the serious causes of disease in small ruminants resulting in economic losses [19]. We performed transcriptome analysis using RNA-seq to identify related genes and pathways that are immediately relevant for the improvement of goat immunity, taking advantage of our resident goat herd, developed through selective breeding. We selected the resistant and susceptible YWGs on the basis of their EPG value and pedigree records. Resistant goats always showed lower EPG and higher body weight gain during the browsing period (see Figure 1 and Table 1). The higher body weight gain in resistant goats was due to their ability to cope with the internal nematode infection compared to susceptible goats. There is no evidence for resistant breeds losing their relative advantage compared with those that are more susceptible [20]. Amarante et al. [21] also showed that worm burden and body weight gain of sheep has negative correlation, and that more resistant animals are more productive. In contrast of EPG, weight gain status also has its own significance effect for the evaluation of resistance or susceptibility status of small ruminants, as was implicated in previous studies [21,22,23,24]. Indeed, the weight gain or loss conditions in the consideration of resistant animals are supposed to depend on each individual’s hereditary potential, production status, and overall sizes Identifying genetic components controlling between-, and within-breed distinctions in nematodes resistance has theoretical and practical implications. Towards this end, numerous efforts have been made over the decades to unravel genes and/or genetic variants responsible for resistance, somewhat driven by strong desires to breed farm animals with strong resistance traits. The two groups of goats have showed distinct differences in GIN resistance (see Table 1 and Figure 1), even when we allowed them to co-graze together on the same pasture under natural conditions [25]. Under artificial challenge trial with *Haemonchus contortus*, we observed that resistant goats had lower FECs than the susceptible goats, by approximately 50%, similar to previous studies [25,26]. Additionally, resistant YWGs not only shed fewer GIN eggs, but also tended to have a delayed egg production, indicating an anti-fecundity effect of the immune response in this selected group. The challenge trial also showed that the days of the first *Haemonchus contortus* eggs were found in the feces from resistant goats later than that of the susceptible goats, which agreed with the study of Gonzalez et al. [25].

We measured blood parameters every alternate month of the browsing period, and the findings of blood parameters revealed significant associations with resistant and susceptible goats, especially in regards to PCV and hemoglobin (see Table 1), which could be used as a valuable means of identifying whether breeds have resistance against internal nematodes. Practically, resistant animals showed low FEC and high PCV. These criterions are keys to assessment of the genetic resistance of an individual against GIN infections. The statistical heritability estimates are usually used based on these criterions for finding resistance within breeds. Among different immune cells, higher number granulocytes (eosinophil, basophil, and neutrophil) were found in resistant group than in susceptible group (see Table 1), suggesting that resistant goats might have developed capabilities for improved recruitment of granulocytes to the place of infection. In these two studies (natural and experimental infections), resistant and susceptible goats showed distinct differences in parasitological and hematological parameters. Resistant goats tend to have considerably reduced nematodes burden, reduced egg laying, and decreased EPG in feces than susceptible goats. Based on these two studies, eight goats were selected for transcriptome analysis.

The transcriptomic analysis of RNA-seq showed that 2369 genes were sufficiently transcribed, of which were 1407 up-regulated and 962 down-regulated and were significantly (*p* < 0.001) differentially expressed between studied groups (see NCBI Sequence Read Archive (SRA); Bio Project ID: PRJNA 341785) that were developed through selective breeding. Functional annotation of these DEGs was highly expressed in the resistant group, yielding 46 significant (*p* < 0.05) clusters, including 31 genes (9 in innate immunity, 13 in immunity, and 9 in innate immune response) related to immune biosynthetic process (see Table 3). Among the DEGs amid abundant transcripts of peripheral blood of selected resistant goats, a remarkable feature was the down-regulation in susceptible goats of various signaling components such as chemokine (C-C motif), interferon, α-inducible protein-6, interferon-induced protein 44-like, and leukocyte specific transcript-1. In order to achieve improvement in the resistance of goats to GINs, our findings propose that immune signaling components may play a vital role in invoking efficient immune responses. Complement activation is one of the most primitive events in host immune responses to GIN infections [10]. Complement related genes were significantly impacted by infection in the susceptible goats compared to uninfected resistant goats, while none of these genes were affected by infection in the susceptible breed. As a result, complement pathways appeared to be activated in the resistant breed.

We performed KEGG pathway analysis, and most of the pathways were also immune response related. The greatest number of genes were related with pathways in endocytosis (see Table 4). Endocytosis related genes play important roles during the wound repair stages, inflammation, formation of new tissues, and remodeling, all of which are highly related with the GIN infections in animals [27]. Gasbarre [28] acknowledged the important roles of Th2 cytokines in preventive tissue damage consequences of infections in rodent models, particularly the involvement of IL1R2 in the early tissue repair stage through its function in neutrophil recruitment. GIN infections, especially *Haemonchus contortus* infections, generally induce a potent T helper type-2 (Th2) immune response in small ruminants [29]. We observed up-regulation of Th2 cytokines in resistant goats. The Th2 immune responses in small ruminants are characterized by the recruitment and activation of mast cells, basophiles, and eosinophils, and goblet cell hyperplasia in airway and intestinal epithelia [30,31]. These immune responses are induced against GINs assaulting cutaneous or mucosal sites and work as protective immunity against those infections. Interestingly, Koyasu et al. [32] showed that that cytokines are induced soon after GIN infection and before pathogen-specific Th2 cells are established. The resistant small ruminants might have developed abilities for enhanced recruitment of these immune cells to the site of infection and resistant individuals are capable of rapidly up-regulating the Th2 cytokines in comparison to the susceptible individuals [33].

A number of genes that impacted resistant goats in our study were related to the MAPK signaling pathway (see Table 4), which regulates translation and transcription. This pathway plays a role in immune activation, as shown for orthologues in vertebrates during a *Coxiella burnetii* infection. *Caenorhabditis elegans* daf-2(–) mutants are hyper-immune and exhibited significantly reduced pathological consequences during challenge [34]. Furthermore, a number of genes were involved in transforming growth factor beta (TGF-β) signaling pathway (see Table 4). TGF-β is a multifunctional cytokine belonging to the transforming growth factor super family, which includes three different isoforms (TGF-β 1 to 3, HGNC symbols TGFβ1, TGFβ2, TGFβ3) and many other signaling proteins produced by all WBC lineages that are TGF-β signaling pathway related [35]. We also found some genes that were related with cell adhesion molecules (CAMs) pathways. CAMs are proteins located on the cell surface [36] involved in binding with other cells or with the extracellular matrix (ECM). It provides essential links between the extracellular environment and the intracellular signaling pathways, which can play important roles in cell behaviors related with the infections, such as apoptosis, differentiation, and survival [37].

In this study, the highest number of genes were enriched in the genetic association disease classes, including ATPase, Cu++ transporting, α polypeptide, B-cell CLL, lymphoma-2, integrin α4, ST6 β-galactosamide α-2,6-sialyltranferase-1, and chemokine-like receptor 1, in selected susceptible YWGs (see Figure 4 and Figure S2). A chromosomal aberration involving BCL2 may be a cause of follicular lymphoma, also known as type II chronic lymphatic leukemia. It regulates cell death by controlling the mitochondrial membrane permeability and appears to function in a feedback loop system [38]. An overall appraisal of transcript abundance of DEGs showed KEGG and GO enrichment related to the immune system. At least four enriched signaling pathways identified under this study (i.e., endocytosis, MAPK signaling pathway, cell adhesion molecules, and TGF-β signaling pathway) are intimately associated with GIN infections. However, the exact relationship between these signaling networks is not fully understood. Some up- and down-regulated DEGs that had not been reported to be involved in resistance to GIN infections were identified, namely complement C3 precursor, integrin α4, B-cell CLL/lymphoma 2, leukocyte specific transcript 1, interferon-induced protein 44-like, and interleukin 1 receptor type II. Our results thus provide new information on resistance mechanisms to GIN infection in YWGs.

## 4. Materials and Methods

### 4.1. Animal Selection from Breeding Herd of Yichang White Goats (YWGs)

This research was performed in strict accordance with the guidelines for experimental animals established by the Ministry of Science and Technology. All the experimental methodology and research on animals were conducted according to the regulations (No. 5 proclaim of the Standing Committee of Hubei People’s Congress) approved by the Standing Committee of Hubei People’s Congress, and the ethics committee of Huazhong Agricultural University, Wuhan, China (Permission number: 4200896859).

A total of 122 resistant (low EPG, *n* = 48) and susceptible (high EPG, *n* = 74) goats were selected from *Yichang* White Goats (YWGs) herd (having more than 800 goats) by selective breeding on the basis of high and low EPG (eggs per gram) of feces from *Lao Gao Huang* goat farm. Once the breeding female goats (high or low EPG) were identified, then semen from known bucks (high or low EPG) were used to produce offspring of desired phenotypes. Then these offspring were kept with their dams following intensive rearing system with minimum or no exposure to parasitic infection from birth to weaning. After weaning of the existing group at 120 ± 15 days of age, offspring were reared under a semi-intensive system up to one year of age. Then, these goats were allowed to browse on infected areas following an extensive rearing system and were monitored fortnightly for a number of parasitological and hematological parameters (four times, every alternate month) along with their growth rate from April to October 2015. This experiment resulted in a resource population selected for the EPG trait (low EPG represents resistant and high EPG represents susceptible). Based on fortnightly EPG counts and pedigree records (i.e., EPD (Expected Offspring/Progeny Difference) assessments), eight goats at 575 ± 15 days of age were selected for artificial challenge trial and transcriptome analysis. Out of the selected eight goats, four goats were defined as selected resistant (low FEC, EPD value) and another four were defined as susceptible (high FEC, EPD value).

### 4.2. Phenotypic Data Collection

All of the selected goats (*n* = 122) were monitored fortnightly for parasitological (EPG) and hematological parameters (hemoglobin, PCV, RBC, and WBC, including lymphocyte, monocyte, and granulocyte) along with weight gain or loss during the period in which they were browsing on an infected area. From each experimental animal, fecal samples were collected from their rectum, and peripheral blood samples were drawn aseptically from the jugular vein. FEC were determined by using modified McMaster technique [39] and different blood parameters were determined using Mindray (BC-2800Vet) Auto Hematology Analyzer (Shenzhen Mindray Bio-Medical Electronics Co. Ltd., Shenzhen, China).

### 4.3. Nematode Challenge Trial

Nematode challenge trial was also performed in the *Lao Gao Huang* goat farm, Yichang, China. The eight selected goats that were 575 ± 15 days of age were used for this artificial challenge trial. At the end of the browsing period, all goats were treated with ivermectin to eradicate GIN infections. We checked and found a few individuals that still showed positive for GINs on fecal examination 15 days after ivermectin treatment; these individuals were further treated by levamisole. We then did fecal examinations again and found an absence of GIN eggs. The goats were fed clean grasses (*Pennesetum purpureum* and *Brachiaria mutica*) cut and carried from fenced fodder land, which were not allowed to be grazed upon by any other animals, and were determined to be GIN infection free. Concentrate diets were also fed twice per day to the animals at the rate of 1.5% body weights. The concentrate diets were composed of broken corn, sesame oil cake, soybean meal, crushed oyster shell, di-calcium phosphate, and salt. The concentrate diet had 16.5% CP (crude protein). Clean and safe water were provided ad libitum to the experimental goats. The feeding and watering pen was cleaned every day to avoid chance of auto infections. The third stage (L3) larvae of *Haemonchus contortus* were isolated from other infected YWGs. The viability of L3 larvae was determined by microscopic examination before every dosing. The larvae were fed to the tested goats orally by using a syringe. The goats were fed 735 larvae of *Haemonchus contortus* every alternate day up to a total of seven doses with 5145 L3 given per animal of resistant and susceptible groups. Feces of experimental goats were checked throughout the trial period for the first signs of eggs of *Haemonchus contortus*. Based on the timing of positive confirmation for *Haemonchus contortus* eggs among all the experimental goats, the experiment lasted for six weeks after all doses were administered. Weight gain or losses of goats were also monitored throughout the artificial challenge trial period. At day 42 of artificial challenge trial, experimental goats (four resistant and four susceptible) were sacrificed for worm counts.

### 4.4. Worm Counts at Necropsy

After slaughter of the experimental resistant and susceptible goats, the abomasum and small intestine was separated, opened, and its contents were taken into a container. The abomasal and intestinal mucosa was thoroughly washed, with the washings added to the contents. Then, 10% of the contents was washed and searched for nematodes. After completion, another 10% of the contents was examined, and all worms were one by one counted from all contents collected from the abomasum as well as small intestine.

### 4.5. Extraction of RNA and Sequencing Using RNA-Seq Technology

Experimental total RNA for sequencing and qPCR analysis was extracted from peripheral blood using the TRIzol Reagent (Invitrogen, Carlsbad, CA, USA) according to the manufacturer’s instructions. The quality and concentration of RNA was detected by Nano Drop ND2000 (Thermo Fisher Scientific, Waltham, MA, USA) spectrophotometer and gel electrophoresis. Next generation sequencing technology was used for this experiment to detect the DEGs in the resistant and susceptible goats. A total of eight cDNA libraries were constructed using the TruSeq Stranded Total RNA LT Sample Prep Kit (Illumina, Santiago, CA, USA). The library construction was performed by Genergy biological technology limited company (Shanghai, China). The metadata and raw sequences data files (eight files) related to this experiment were deposited in the NCBI Sequence Read Archive (SRA) (Bio project ID: PRJNA 341785, accession numbers SRX2181195, SRX2181163, SRX2181139, SRX2181120, SRX2181119, SRX2181118, SRX2181117, and SRX2181116).

### 4.6. Quality Control for Raw Sequencing Data

The data of RNA sequencing were received from the sequencing facility in FASTQ files that are exemplified tool command lines using a paired-end formatted file set [40]. Then we checked the overall sequence quality and the data with clean reads were obtained by trimming the adapter contaminants and filtering the low-quality reads. A quality control tool, Trimmomatic [41], which can perform various useful forms of trimming was utilized for the quality control of our raw sequence data.

### 4.7. Transcriptome Analysis for mRNA Data

The reference genomes of *Capra hircus* (CHI_1.0) and the gene annotation information downloaded from the NCBI database (available at: ftp://ftp.ncbi.nlm.nih.gov/genomes/Capra_hircus/) were used in this analysis. After filtration by Trimmomatic, all clean reads of each sample were mapped to the reference sequence with TopHat-2 [42]. Subsequently, Cuffdiff were used to calculate gene and transcript expression in each sample following FPKM (fragments per kilobase of transcript per million mapped fragments) method to calculate gene expression [43]. In addition, to get an overview of gene expression difference between these two groups, a standardized reads-count of HTseq was fit to gplots to produce the heatmap.

To gain insight into the function of the DEGs in the resistant and susceptible YWGs with the following parameters: Count = 2 and EASE = 0.01, DAVID (Database for Annotation, Visualization and Integrated Discovery) (available at: http://david.abcc.ncifcrf.gov/) [44], bioinformatics resource tools were used for gene annotation and pathway analysis. *Homo sapiens* were used as a reference species due to the lack of data for *Capra hircus* in the DAVID website. The functions of genes in biological processes, cellular components, and molecular processes were annotated based on the GO categories.

### 4.8. qRT-PCR for Validation of DEGs

The RNA sequencing data were validated through qRT-PCR, using a standard Prime Scipt^TM^ RT reagent Kit with gDNA eraser (Perfect Real Time), TAKARA Bio Inc. for cDNA production, and SYBR Green real time PCR master mix (Toyobo Co., Ltd., Osaka, Japan) for qRT-PCR reaction following manufacturers’ instructions. Twelve up- and down-regulated DEGs from Table 5 were selected for qRT-PCR validation. The primers were designed from NCBI primer blast and primers were checked using OLIGO7 software (Molecular Biology Insights) in accordance with the sequences of the corresponding goat mRNAs in GenBank. A list of primers of selected genes for qRT-PCR experiment is shown in Table S3. Goat β-actin gene was used as a housekeeping gene in this qPCR validation experiments as a control gene for normalization of cDNA loading differences in this experiment. To avoid genomic DNA contamination, 2.0 µL of 5× gDNA eraser buffer, 1.0 µL of gDNA eraser, 1.0 µL of total RNA with a concentration of 1000 ng/µL, and 6.0 µl of RNase free dH_2_O were used to treat the RNA sample per reaction reagent. The reaction reagent of cDNA production contained 10.0 µL of gDNA elimination mix solution, 4.0 µL of 5× Prime Script buffer (for qRT-PCR), 1.0 µL of enzyme, 1.0 µL of RT primer mix, and 4.0 µL of RNase free dH_2_O. The qRT-PCR reaction contained 5.0 µL of SYBR Green real time PCR master mix (Toyobo Co., Ltd., Osaka, Japan), 0.2 µL of forward and reverse primer each, 1.0 µL of cDNA, and 3.6 µL of RNase free dH_2_O. The qRT-PCR reaction was performed on a Bio-Rad thermal cycler, CFX-384, real time system as follows: single cycle of denaturation at 95 °C for 5 min, 45 cycles of denaturation at 95 °C for 15 s, annealing at 60 °C for 20 s, and extension at 72 °C for 5 s.

### 4.9. Statistical Analysis

The statistical analyses of the phenotypic significance differences between the resistant and susceptible groups were tested by Student’s *t*-test. The qRT-PCR amplifications were conducted using an independent set of four biological and three technical replicates per sample. Relative quantification analyses were performed using the 2^−ΔΔ*C*T^ value method [45,46]. Comparisons between qPCR datasets were also calculated using Student’s *t*-test, and differences were considered significant when *p* < 0.05.

## 5. Conclusions

Two groups (resistant group and susceptible group) of YWGs displayed a distinct difference in several GIN phenotypes under natural and artificial infections. Resistant goats tended to have notably reduced nematodes burden, delayed first egg laying, and decreased EPG in feces in comparison to the susceptible goats. We accomplished RNA-seq profiling to identify related genes and pathways that appear to be the cause of the improvement of goat’s resistance, by taking advantage of our resident goat herd developed through selective breeding. It is the first transcriptomic detailed investigation in the peripheral blood of YWGs to GIN infections and defines specific genes connected with the goats’ resistance and immune response genes involved in these processes. A wide range of pathways may have evolved in resistant goats to provide protection against GIN infections. Readily inducible immune responses complement activation and innate as well as acquired immunity directly against worm fecundity and are likely to contribute to the improvement of the immunity of the goats to GIN infections in resistant goats. Pathways of TGF-β, MAPK, and CAMs signaling may contribute to disease resistance of YWGs to GINs infection. The results generated by this study have provided information not only on responsible genes but also on possible mechanisms to produce a GIN resistant goat breed. Further immune-histochemistry and physiological study of these candidate genes will be conducted in the future.

## Figures and Tables

**Table 1:** Weight gain, egg per gram (EPG), and blood parameters comparison of low EPG offspring group and high EPG offspring group of *Yichang* White Goats (YWGs) (*n* = 122).

Parameters	Low EPG Offspring Group	High EPG Offspring Group
(a) EPG (fortnightly mean)	11.43 ± 1.81 *	42.26 ± 6.56
(b) Live-weight gain (g/day)	34.75 ± 2.65 *	20.16 ± 2.38
(c) Blood parameters		
1. Hemoglobin (g/L)	104.58 ± 1.37 *	89.62 ± 1.14
2. Packed cell volume, PCV (%)	30.11 ± 0.48 **	28.04 ± 0.41
3. Red blood cells, RBCs (10^12^/L)	10.60 ± 0.32	10.08 ± 0.31
4. White blood cells, WBCs (10^9^/L)	10.70 ± 0.82	10.81 ± 0.56
5. Lymphocytes (10^9^/L)	4.53 ± 0.71	4.24 ± 0.43
6. Monocytes (10^9^/L)	2.21 ± 0.22	2.24 ± 0.14
7. Granulocytes (10^9^/L)	4.43 ± 0.40	4.33 ± 0.51

Mean ± SEM, * Significant (*p* < 0.01), based on assuming unequal variances Student’s *t*-test. ** Significant (*p* < 0.05), based on assuming unequal variances Student’s *t*-test.

**Table 2:** The summary of the RNA sequencing analysis data from peripheral blood samples of resistant and susceptible goats.

Parameters	Resistant YWGs				Susceptible YWGs			
	(X18)	(A002)	(H095)	(X248)	(A101)	(X066)	(X043)	(B15)
Total mapped reads	30,934,247	30,592,726	27,854,500	34,486,633	35,637,910	36,358,502	42,719,520	43,168,067
Matched reads (A)	29,502,236	29,164,015	26,573,158	32,797,772	33,860,257	34,389,638	40,633,313	40,911,091
Properly Matched reads (B)	23,212,930	23,379,066	21,586,978	27,844,776	30,443,862	30,571,188	35,726,058	35,855,556
Ratio of (B) and (A)	78.68%	80.16%	81.24%	84.90%	89.91%	88.90%	87.92%	87.64%
Singletons	4,668,298	4,298,055	4,155,394	3,969,492	2,260,933	2,363,108	2,700,003	2,501,895

**Table 3:** Major functional annotation clusters (top 10 annotation clusters based on fold change (FC) and *p* < 0.05) in YWGs.

Gene Ontology (GO) Name	Number of Genes Involve	Fold Change	Genes	
			Highly Expressed in Susceptible Group	Highly Expressed in Resistant Group
Negative regulation of transcription from RNA polymerase II promoter	21	2.0	*ELK4*, *GATAD2A*, *KANK2*, *MNT*, *ARHGAP35*, *SMAD3*, *TAL1*, *CBX6*, *GFI1*, *HDAC4*, *NFATC2*, *RXRA*, *RUNX3*, *ZNF8*	*N4BP2L2*, *ANKRD2*, *BMP4*, *DUSP26*, *FOXH1*, *HSF4*, *IRF7*
Ion transport	17	2.0	*ATP7A*, *KCNA1*, *SLC39A10*, *TTYH3*, *TPCN1*	*ATP5E*, *ATP5I*, *FXYD1*, *CACNA2D4*, *CHRNA2*, *KCNK7*, *KCNJ15*, *SLC10A1*, *SLC22A17*, *SLC26A8*
Protein phosphorylation	15	2.2	*PDPK1*, *ATM*, *BCR*, *JAK3*, *SBK1*, *ACVR1B*, *EIF2AK3*, *HUNK*, *LMTK2*, *PRKCI*, *PDK4*, *RUNX3*	*DMPK*, *NTRK1*, *TEX14*
Immunity	13	1.9	*DDX3X*, *ETS1*, *JAK3*, *C3*, *ERAP1*, *LST1*	*CLEC4E*, *ISG15*, *S100A12*, *ZBP1*, *APOBEC3A*, *IRF7*, *PGLYRP1*
Innate immunity	9	2.5	*DDX3X*, *JAK3*, *C3*	*ISG15*, *S100A12*, *ZBP1*, *APOBEC3A*, *IRF7*, *PGLYRP1*
Innate immune response	9	1.4	*DDX3X*, *JAK3*, *APOBEC3A*, *F12*	*CLEC4E*, *S100A12*, *ZBP1*, *IRF7*, *PGLYRP1*
RNA polymerase II regulatory region seq-specific DNA binding	9	2.9	*GATAD2A*, *MNT*, *PRDM15*, *SMAD3*, *TAL1*, *RXRA*, *RUNX3*, *ZNF8*	-
GTPase activation	9	3.4	*AGAP1*, *ADAP1*, *BCR*, *ARHGAP23*, *ARHGAP35*, *TBC1D2B*, *TBC1D9*, *SIPA1L3*	*TBCD7*
Peptidyl-serine phosphorylation	8	4.4	*PDPK1*, *ATM*, *BCL2*, *RICTOR*, *EIF2AK3*, *LMTK2*, *PRKCI*	*DMPK*
Rab GTPase binding	6	3.0	*ERC1*, *RAB11FIP4*, *RAB29*, *TBC1D2B*, *TBC1D9*	*TBC1D7*

**Table 4:** The Kyoto Encyclopedia of Genes and Genomes (KEGG) pathway regulated by DEGs of resistant and susceptible YWGs.

Gene Ontology (GO) Accession	Sub-Pathways in KEGG	Gene Count	Genes
hsa04144	Endocytosis	8	*RAB11FIP4*, *ACVR1B*, *PARD3*, *NTRK1*, *RAB5A*, *PRKCI*, *AGAP1*, *IQSEC2*
hsa05016	Huntington’s disease	6	*ATP5E*, *TAF4*, *POLR2L*, *NDUFA1*, *NDUFB1*, *HIP1*
hsa04010	Mitogen-activated protein kinase (MAPK)	6	*IL1R2*, *ACVR1B*, *ELK4*, *NTRK1*, *NFATC2*, *CACNA2D4*
hsa05220	Chronic myeloid leukemia	4	*ACVR1B*, *BCR*, *GAB2*, *SMAD3*
hsa04520	Adherens junction	4	*ACVR1B*, *PARD3*, *TJP1*, *SMAD3*
hsa05410	Hypertrophic cardiomyopathy (HCM)	4	*MYBPC3*, *ITGA4*, *TPM2*, *CACNA2D4*
hsa04350	Transforming growth factor-β (TGF-β)	4	*BMP4*, *ACVR1B*, *RBL2*, *SMAD3*
hsa04514	Cell adhesion molecules (CAMs)	4	*SELP*, *NLGN2*, *L1CAM*, *ITGA4*

**Table 5:** DEGs that are regulated in the immune response of resistant and susceptible YWGs.

Gene IDs	Gene	Official Full Name	Fold Change (FC)			*p*-Value
			(Resistant)	(Susceptible)	(log2)	
XLOC_031254	*ATP7A*	ATPase, Cu^2+^ transporting, α polypeptide	0.094044	1.55104	4.04376	0.00005
XLOC_027074	*BCL2*	B-cell CLL/lymphoma 2	0.44247	9.05593	4.35521	0.00005
XLOC_006794	*CLEC4E*	C-type lectin domain family 4, member E	42.6889	8.79995	−2.27829	0.00005
XLOC_001898	*ITGA4*	Integrin α4 (antigenCD49D, α4 subunit of VLA-4 receptor)	0.887292	37.3364	5.39503	0.0006
XLOC_000758	*ST6GAL1*	ST6 β-galactosamide α-2,6-sialyltranferase 1	1.13434	15.1367	3.73813	0.00005
XLOC_010716	*CCL27*	Chemokine (C-C motif) ligand 27	0.763246	0.122027	−2.64494	0.0069
XLOC_019587	*CMKLR1*	Chemokine-like receptor 1	2.12761	14.6832	2.78686	0.00005
XLOC_009364	*F12*	Coagulation factor XII (Hageman factor)	5.95661	0.791705	−2.91146	0.0001
XLOC_009670	*ERAP1*	Endoplasmic reticulum aminopeptidase 1	1.80804	25.1042	3.79543	0.00125
XLOC_001743	*IFI6*	Interferon, α-inducible protein 6	1535.19	316.823	−2.27667	0.00015
XLOC_003925	*IFI44L*	Interferon-induced protein 44-like	129.911	25.7782	−2.3333	0.00005
XLOC_013178	*IL1R2*	Interleukin 1 receptor, type II	14.3276	2.97729	−2.26672	0.00005
XLOC_026289	*LST1*	Leukocyte specific transcript 1	3019.33	477.558	−2.66048	0.00005
XLOC_021342	*PGLYRP1*	Peptidoglycan recognition protein 1	884.008	215.399	−2.03705	0.00005
XLOC_008453	*C3*	Complement C3 precursor; complement component 3	3.33294	161.046	5.59454	0.00005

## Supplementary Materials

Supplementary materials can be found at www.mdpi.com/1422-0067/18/4/751/s1.
