# Supplementary material for: Exploring the Genetic Resistance to Gastrointestinal Nematodes Infection in Goat Using RNA-Sequencing
Source: Int J Mol Sci. 2017 Apr 1;18(4):751. doi: 10.3390/ijms18040751 (PMC5412336; doi:10.3390/ijms18040751)
Supplement: Supplementary file 1 [file ijms-18-00751-s001.zip › ijms-176018-supplementary files/S 1-Supplementary figure.pdf]

Supplementary figure 1: Fast QC (quality control) data

Goat ID	Basic statistics		Goat ID	Basic statistics	
X18	**Measure**	**Value**	A101	**Measure**	**Value**
	Filename	18_1.paired.fq		Filename	A101_1.paired.fq
	File type	Conventional base calls		File type	Conventional base calls
	Encoding	Sanger / Illumina 1.9		Encoding	Sanger / Illumina 1.9
	Total Sequences	18344612		Total Sequences	22740578
	Filtered Sequences	0		Filtered Sequences	0
	Sequence length	50-130		Sequence length	50-105
	%GC	52		%GC	55
A002	**Measure**	**Value**	X066	**Measure**	**Value**
	Filename	A002_1.paired.fq		Filename	066_1.paired.fq
	File type	Conventional base calls		File type	Conventional base calls
	Encoding	Sanger / Illumina 1.9		Encoding	Sanger / Illumina 1.9
	Total Sequences	20611561		Total Sequences	22153023
	Filtered Sequences	0		Filtered Sequences	0
	Sequence length	50-130		Sequence length	50-105
	%GC	51		%GC	52
H095	**Measure**	**Value**	X043	**Measure**	**Value**
	Filename	H096_1.paired.fq		Filename	043_1.paired.fq
	File type	Conventional base calls		File type	Conventional base calls
	Encoding	Sanger / Illumina 1.9		Encoding	Sanger / Illumina 1.9
	Total Sequences	19275680		Total Sequences	25440163
	Filtered Sequences	0		Filtered Sequences	0
	Sequence length	50-130		Sequence length	50-105
	%GC	51		%GC	50
X248	**Measure**	**Value**	B15	**Measure**	**Value**
	Filename	248_1.paired.fq		Filename	B15_1.paired.fq
	File type	Conventional base calls		File type	Conventional base calls
	Encoding	Sanger / Illumina 1.9		Encoding	Sanger / Illumina 1.9
	Total Sequences	22221239		Total Sequences	25789160
	Filtered Sequences	0		Filtered Sequences	0
	Sequence length	50-130		Sequence length	50-105
	%GC	51		%GC	50

Goat ID	Per base sequence quality	Goat ID	Per base sequence quality

X18

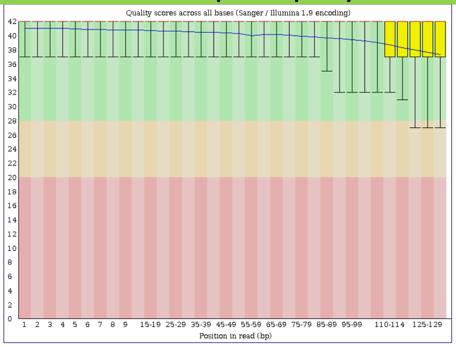

A101

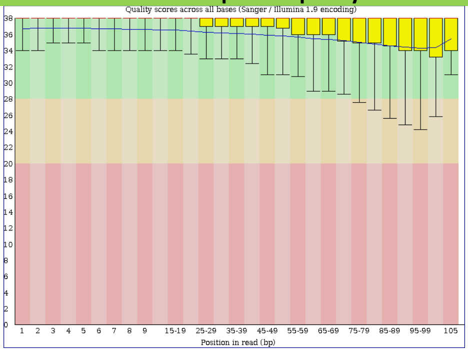

A002

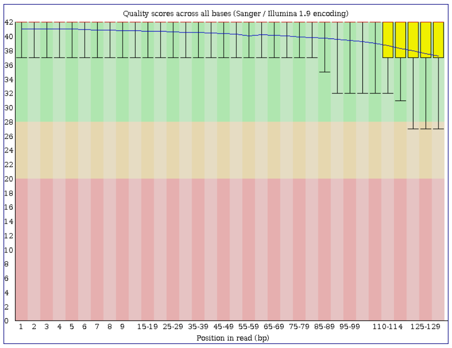

X066

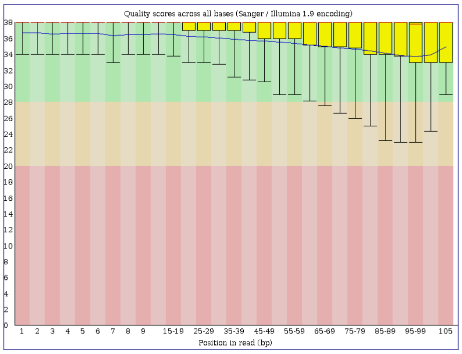

H095

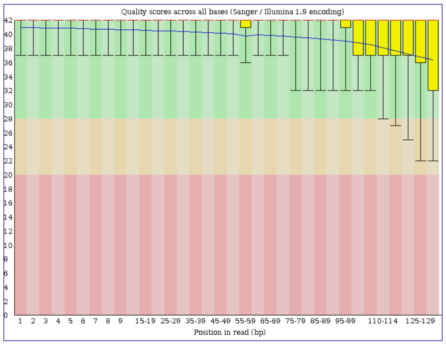

X043

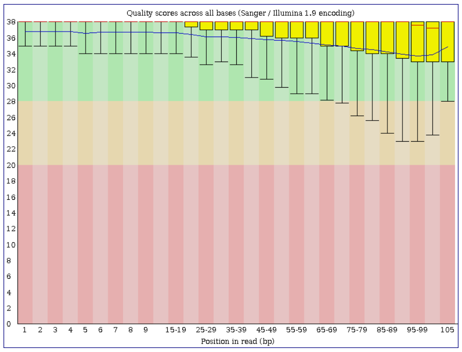

X248

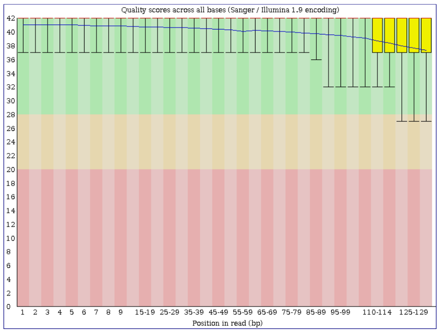

B15

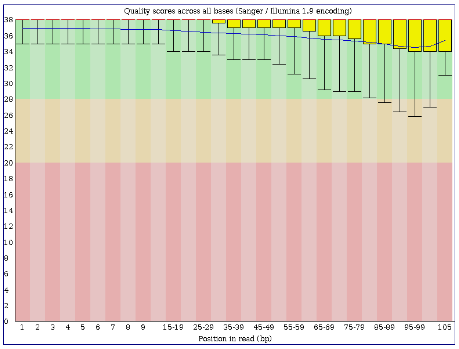

Goat ID	Per sequence quality scores	Goat ID	Per sequence quality scores
X18	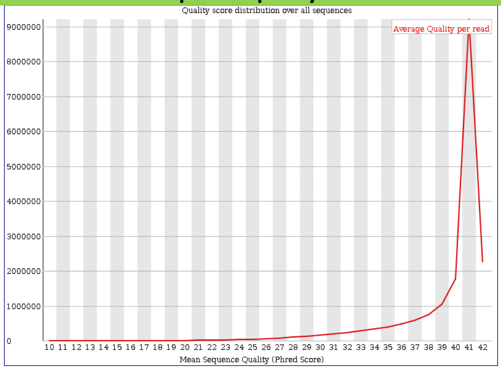	A101	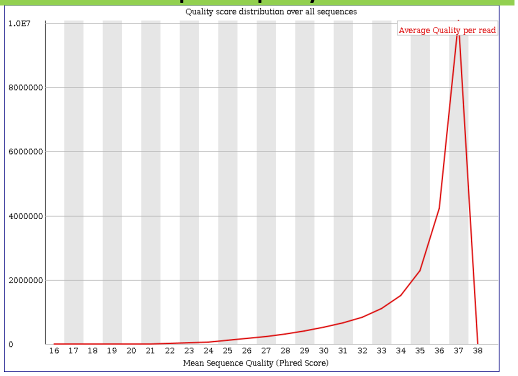
A002	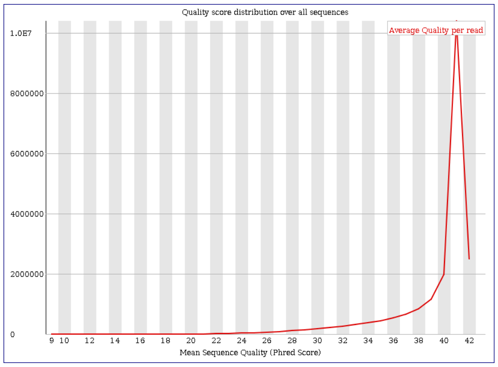	X066	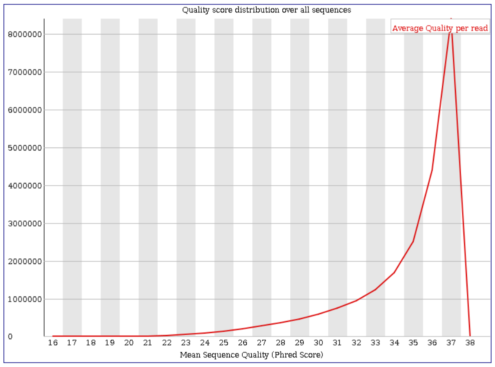
H095	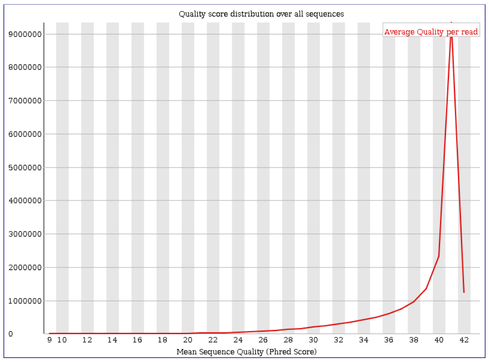	X043	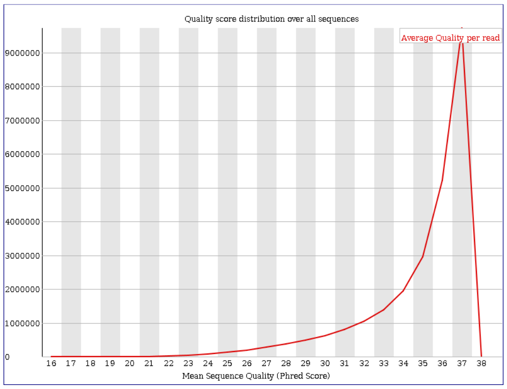
X248	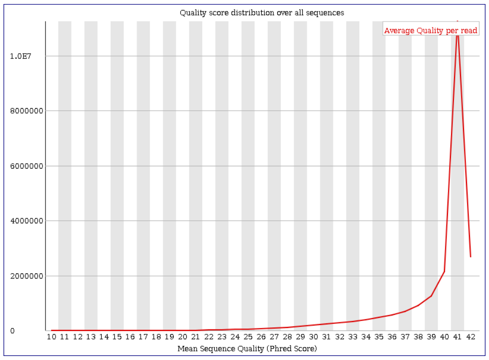	B15	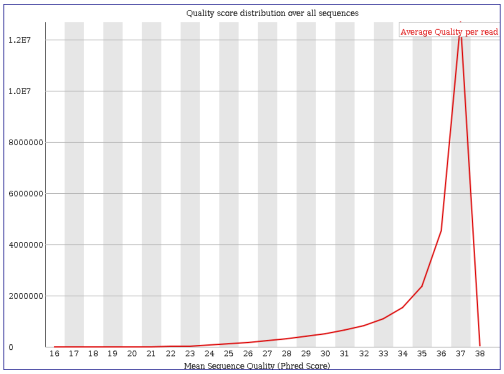

Goat ID | **Per base sequence content** | **Goat ID** | **Per base sequence content**

X18

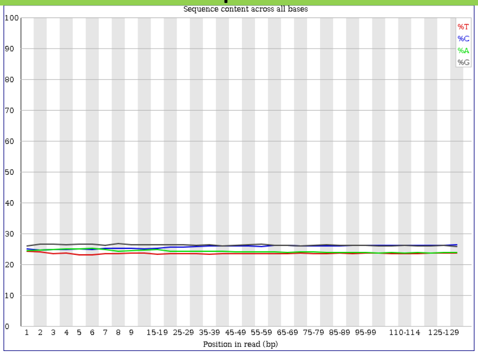

A101

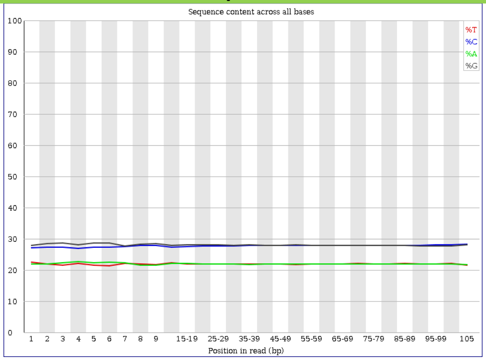

A002

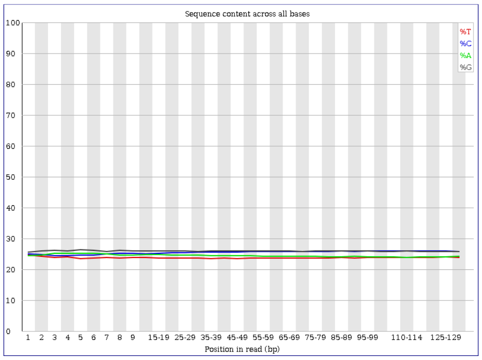

X066

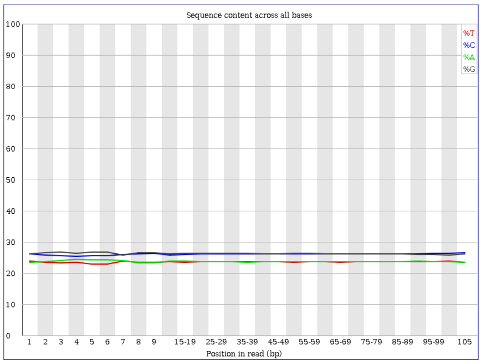

H095

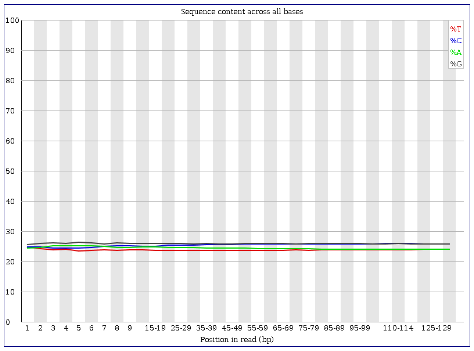

X043

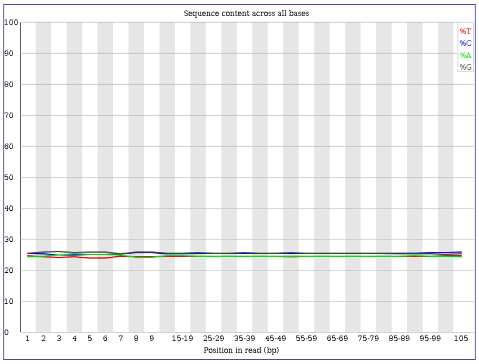

X248

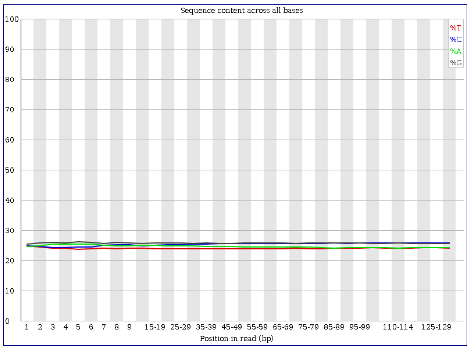

B15

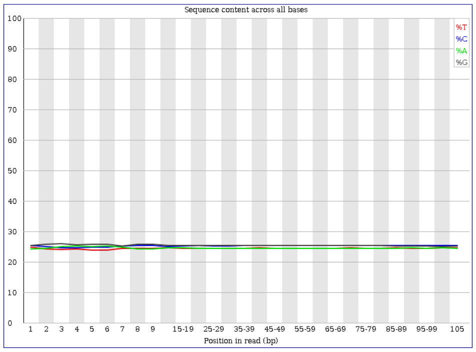

Goat ID	Per base GC content	Goat ID	Per base GC content
**X18**	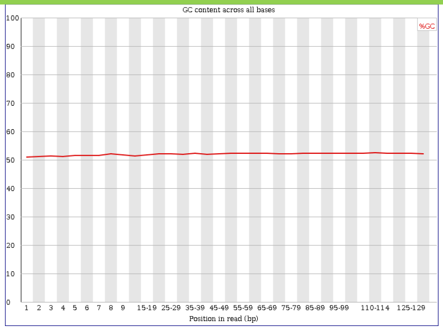	**A101**	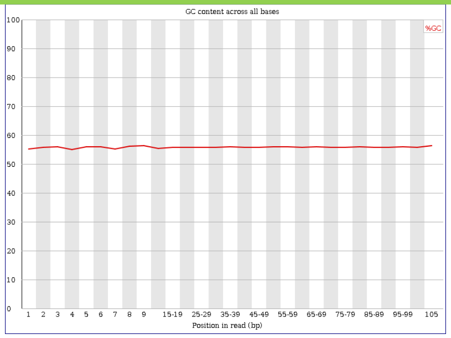
**A002**	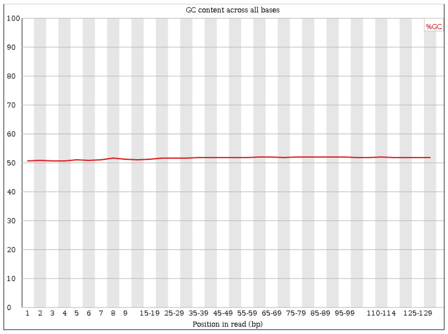	**X066**	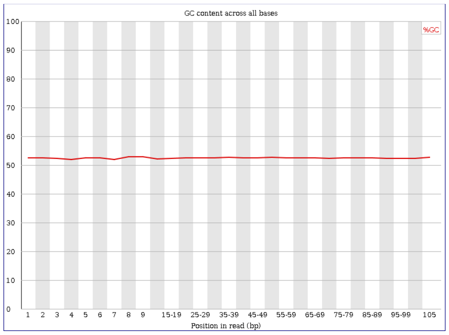
**H095**	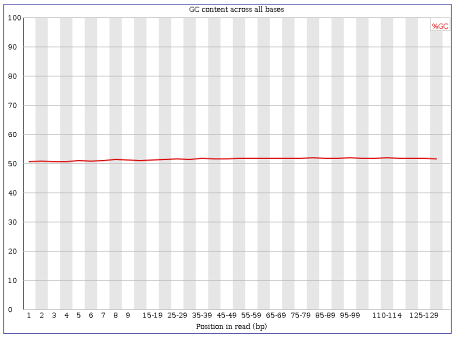	**X043**	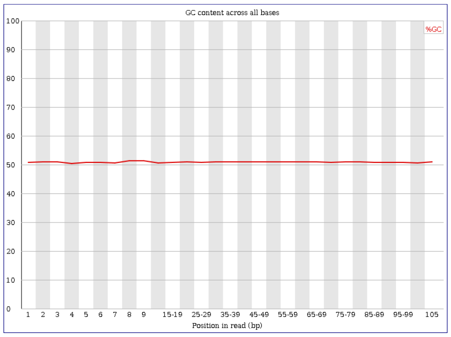
**X248**	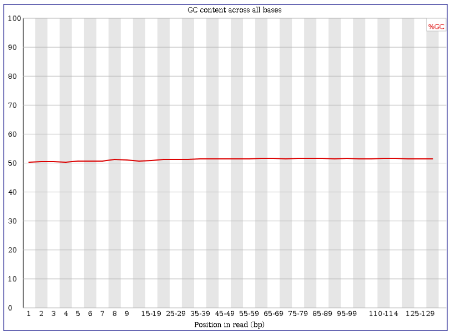	**B15**	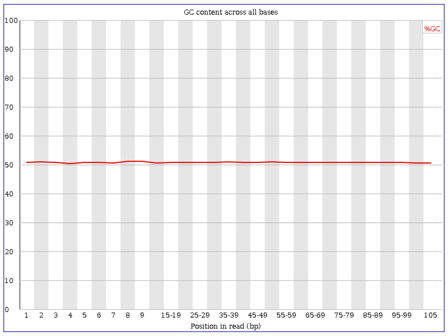
