# Supplementary material for: Exploring the Genetic Resistance to Gastrointestinal Nematodes Infection in Goat Using RNA-Sequencing
Source: Int J Mol Sci. 2017 Apr 1;18(4):751. doi: 10.3390/ijms18040751 (PMC5412336; doi:10.3390/ijms18040751)
Supplement: Supplementary file 1 [file ijms-18-00751-s001.zip › ijms-176018-supplementary files/S 3-Supplementary table.pdf]

Supplementary table 3: List of target genes primer sequences

Genes	Primers	Sequences	Base	GC%	Product length	n.mol/OD	MW
C3	Forward	5' CACCATGCAGCCCACCT 3'	17	64.71	274	6.5	5060.3
	Reverse	5' CTTGATGGTGACGGTGCTCA 3'	20	56.00		5.4	6164.1
ITGA4	Forward	5' CAGTTGGTGCTTTTCGGTCTG 3'	21	52.38	247	5.1	6109.0
	Reverse	5' GACGGAGACTCTGCCTTTCG 3'	20	60.00		5.4	6466.3
BCL2	Forward	5' GGATGACCGAGTACCTGAACC 3'	21	57.14	269	5.1	6182.1
	Reverse	5' CAGCAGTGGCAATGTGGACT 3'	20	56.00		5.3	6182.1
ATP7A	Forward	5' AAGATGAAGGTGGAAGGGATGAC 3'	23	47.83	181	4.6	7250.8
	Reverse	5' AGCCCACAACTTCAATCTGCTT 3'	22	45.45		5.0	6614.4
ERAP1	Forward	5' ACTTTCGCTTTCACTTTTGGTCC 3'	23	43.48	274	4.8	6922.6
	Reverse	5' GCTGGGATGATGTGCTCTGG 3'	20	50.00		5.3	6220.1
ST6GAL1	Forward	5' ACCTTTCAAACCTCAGCTCCG 3'	21	52.38	275	5.2	6286.1
	Reverse	5' GAAAGATGAGCACACAGCAGC 3'	21	52.38		5.1	6482.3
LST1	Forward	5' ATGTCTGAGGCGGCAACAC 3'	19	57.89	181	5.7	5837.8
	Reverse	5' CCAGAACGACCACAAGCAGA 3'	20	56.00		5.4	6098.0
IFI44L	Forward	5' TAGTCCATAAGTGTGACGGAGC 3'	22	50.00	216	4.9	6799.5
	Reverse	5' CCTTGACAACGGCACCTATTCT 3'	22	50.00		5.0	6630.4
IL1R2	Forward	5' AACAACACCCGCATAGAGGAT 3'	21	47.62	294	5.1	6417.2
	Reverse	5' GCGTTTATACCGTCTGTGCATC 3'	22	50.00		4.9	6692.4
F12	Forward	5' GCCCCAGTTTTTCCGGTTCT 3'	20	55.00	255	5.5	6026.0
	Reverse	5' CAGCTCAGTTGGTCGCCTT 3'	19	57.89		5.7	5770.8
CCL27	Forward	5' TGTGGCGGGATGATTGTGAA 3'	20	50.00	273	5.3	6098.0
	Reverse	5' CCGTTTTCCATGGGCTCCTA 3'	20	56.00		5.5	6268.2
IFI6	Forward	5' TCTCTGCTCTCCTCCAAGTTCT 3'	22	50.00	180	5.0	6630.4
	Reverse	5' GTAGCAGGTAGCACAGGAACAG 3'	22	54.55		4.8	6563.3
β-actin	Forward	5' GCAAGGACCTTTACGCCAAC 3'	20	55.00	116	5.4	6204.1
	Reverse	5' CTTGATCTTCATCGTGCTGGG 3'	21	52.40		5.1	6022.0
